# The role of the electrokinetic charge of neurotrophis-based nanocarriers: protein distribution, toxicity, and oxidative stress in in vitro setting

**DOI:** 10.1186/s12951-021-00984-4

**Published:** 2021-08-28

**Authors:** Maria Dąbkowska, Zofia Ulańczyk, Karolina Łuczkowska, Dorota Rogińska, Anna Sobuś, Monika Wasilewska, Maria Olszewska, Katarzyna Jakubowska, Bogusław Machaliński

**Affiliations:** 1 Department of Medical Chemistry, Pomeranian Medical University, Rybacka 1, 70-204 Szczecin, Poland; 2 Department of General Pathology, Pomeranian Medical University, Rybacka 1, 70-204 Szczecin, Poland; 3 Jerzy Haber Institute of Catalysis and Surface Chemistry Polish Academy of Sciences, Niezapominajek 8, 30-239 Cracow, Poland; 4 Department of Biochemistry, Pomeranian Medical University, Rybacka 1, 70-204 Szczecin, Poland

## Abstract

**Background:**

The rational chemical design of nanoparticles can be readily controlled and optimized by quantitatively studying protein adsorption at variously charged polymer carriers, determining their fate in biological fluids. We manufactured brain-derived neurotrophic factor (BDNF) -based electrostatic nanocomplexes with a different type of dendrimer core (anionic or cationic), encapsulated or not in polyethylene glycol (PEG), and studied their physicochemical properties and behavior in a biological setting. We investigated whether the electrokinetic charge of dendrimer core influences BDNF loading and desorption from the nanoparticle and serves as a determinant of nanoparticles’ behavior in in vitro setting, influencing mitochondrial dysfunction, lipid peroxidation, and general nanoparticles’ cellular toxicity.

**Results:**

We found that the electrokinetic charge of the dendrimer core influences nanoparticles in terms of BDNF release profile from their surfaces and their effect on cell viability, mitochondrial membrane potential, cell phenotype, and induction of oxidative stress. The electrostatic interaction of positively charged core of nanoparticles with cell membranes increases their cytotoxicity, as well as serious phenotype alterations compared to negatively charged nanoparticles core in neuron-like differentiated human neuroblastoma cells. Moreover, PEG adsorption at nanoparticles with negatively charged core presents a distinct decrease in metabolic cell activity. On the contrary, charge neutralization due to PEG adsorption on the surface of nanoparticles with positively charged core does not reduce their cytotoxicity, makes them less biocompatible with differentiated cells, and presumably shows non-specific toxicity.

**Conclusions:**

The surface charge transformation after adsorption of protein or polyelectrolyte during nanocarriers formulation has an important role not only in designing nanomaterials with potent neuroprotective and neuroregenerative properties but also in applying them in a cellular environment.

**Graphic abstract:**

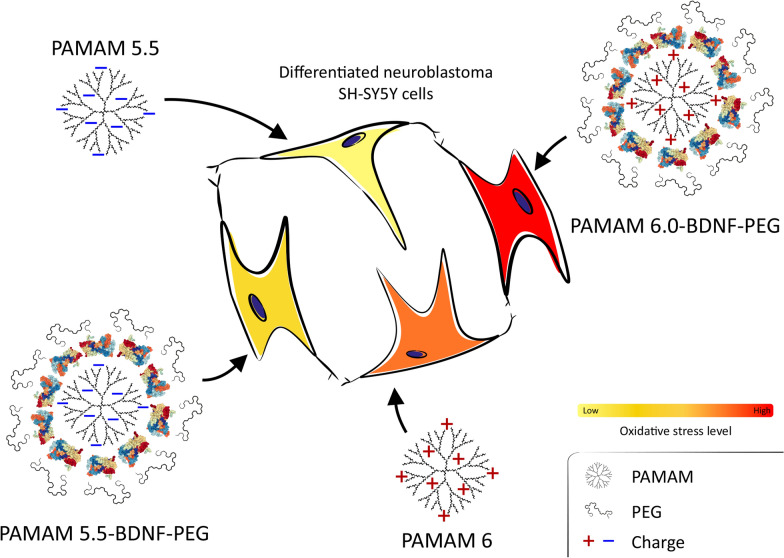

**Supplementary Information:**

The online version contains supplementary material available at 10.1186/s12951-021-00984-4.

## Background

Recent nanotechnology advancements have enabled the introduction of nanoparticles (NPs) with potential applications in various areas; in the medical field, they are used as delivery systems for drugs, proteins, and DNA [1, 2]. To date, NPs have been constructed from various substances, including metal and non-metal, polymeric, and bioceramic materials [3]. Liposomes, polyethylene glycol (PEG), and dendrimers are the main constituents of NPs used for therapeutic applications [4, 5]. Regardless of their chemical composition, protein-loaded NPs should allow efficient loading and retention of the therapeutic protein, offer high stability in the biological setting, and ensure the fast release of protein readily available for its receptors at its desired site of action [6, 7].

It is particularly tempting to utilize protein-loaded NPs in the central nervous system (CNS). The anatomical obstacle in the blood‐brain barrier (BBB) makes free protein delivery to the tissue of interest particularly challenging. Neurotrophins, including brain-derived neurotrophic factor (BDNF), have been interesting therapeutic protein candidates for neurodegenerative disorders, particularly for their neuronal survival-promoting features [8]. The utilization of NPs for neurotrophins delivery to the brain tissue could improve their therapeutic potency by increasing circulation time and bioavailability at the targeted site [9]. However, since general NPs limitation in an in vivo setting, which significantly reduces therapeutic effects of transported proteins, is clearance via the mononuclear phagocytic system [10], neurotrophins-loaded NPs design requires proper understanding of their physicochemical properties that may influence their behavior in the biological milieu.

Polyamidoamine (PAMAM) dendrimers have been considered a promising material for NPs designed for CNS delivery, as they have several beneficial features: high stability and water-solubility, small size, presence of cavities, and modifiable surfaces [11, 12]. In fact, PAMAM dendrimer attributes (core, size of dendrimer/cavities, functional groups on the surface) can be accurately modified and provide a range of NPs for in vitro or in vivo drug delivery to brain cells [13, 14]. Additional PEGylation (PEG modification) of PAMAM surface is a simple and effective way to reduce aggregation, phagocytosis and improve encapsulation, biocompatibility, and targeting of the drug [5, 15]. However, the precise mechanism of how (and even if) the dendrimer enters the cell and how the transported protein is released remains not fully understood [16].

It has been hypothesized that the surface charge of the dendrimer is an important factor in considering NPs effective: it influences cellular uptake, biocompatibility, receptor binding, and toxicity and is also linked with the capacity to produce reactive oxygen species (ROS) [17]. The electrostatic interaction between PAMAM dendrimers with positively charged amino groups and negatively charged cell membrane could lead to significant thinning and disruption of the biological lipid bilayer membrane, including the formation of nano-holes [18, 19]. It has been suggested that positivelycharged NPs show less systemic benefits, as they are more prone to unintended liver uptake, which is generally in concordance with the observation that cationic macromolecules are more rapidly cleared from plasma [20]. On the contrary, Florendo et al. hypothesized that positive charge on dendrimers enhances adsorptive endocytosis and enables more effortless cell membrane crossing [13]. It makes things even more complicated that when desired protein is adsorbed onto the NP surface, it can generate a change in zeta potential, independent of initial surface charge [5, 21]. Since the surface charge affects not only cellular uptake of NPs, but also retention in circulation [16, 22], the precise knowledge on the charge in NPs design is of great importance.

The main goal of our study was to thoroughly characterize PAMAM- and PEGylated PAMAM-based nanoparticles with differently charged PAMAM cores. We investigated whether the electrokinetic charge of PAMAM core in PAMAM-based nanoparticles influences BDNF loading and desorption from the nanoparticle. We also aimed to establish whether PAMAM core charge is a determinant of nanoparticles’ behavior in vitro setting, influencing mitochondrial dysfunction, lipid peroxidation, and general nanoparticles’ cellular toxicity.

## Materials and methods

### Preparation and characterization of PAMAM-based nanoparticles with negative/ positive core:

The suspension of ethylenediamine core five and a half-generation dendrimers (PAMAM G5.5) with sodium carboxylate surface groups, (536784, Sigma Aldrich, St. Louis, MO, USA) as well as ethylenediamine core six-generation dendrimers (PAMAM G6) with amino surface groups (536717, Sigma Aldrich) were used as a colloid carrier for BDNF. Each stock suspension was diluted before each adsorption experiment to the desired mass concentration, equal to 10 mgL^−1^. Filtered (Centrifree ultrafiltration device, Merck Group, Darmstadt, Germany) stock solutions of carrier-free recombinant human BDNF (248-BDB-250/CF, R&D Systems, Canada) of known concentrations (typically 50 mgL^−1^) in the phosphate-buffered saline (PBS) pH 7.4 ± 0.2, 0.15 M (Biomed, Lublin, Poland) were prepared to remove aggregates and provide constant, free form protein molecules concentration before adsorption at PAMAM particles. To minimize errors in concentration measurements, spectrophotometric techniques for the BCA (protein quantification bicinchoninic acid assay, kit for low concentration, Abcam, Cambridge, UK) were used. BDNF adsorption at PAMAM dendrimers was performed employing electrostatic interactions according to the following procedure: (1) the reference of electrophoretic mobility of bare PAMAM nanoparticles was measured, (2) BDNF layers were formed by mixing equal volumes of its solutions of the 0.2 mgL^−1^ bulk concentration, with nanoparticle suspension of the bulk concentration 20 mgL^−1^, (3) the electrophoretic mobility of BDNF-PAMAM nanoparticles was measured and the corresponding zeta potential was calculated. We obtained BDNF-PAMAM nanoparticles, referred to as “PAMAM-based nanoparticles”. Experiments were conducted at 7.4 pH, ionic strength 0.15 M, at room temperature. The whole experimental procedure was performed for an adsorption time of 6000 s. Afterward, BDNF-PAMAM dendrimer nanoparticles were encapsulated with PEG (poly(ethylene glycol)) with a molecular weight of 4 kDa, (1546569, Sigma Aldrich). We carried out simply mixing BDNF-PAMAM and PEG in aqueous solutions without sonification or other extensive agitation. PEG chains were conjugated to the nanoparticle surfaces via amide or carboxyl bonds depending on the type of PAMAM core charge, formation between PEG amino groups and PAMAM surface groups. An equal volume of beforehand prepared BDNF-PAMAM and PEG (50 mgL^−1^, pH 7.4, PBS) solutions was prepared by mixing at room temperature for 1 h. Next, PEG-ylated BDNF-PAMAM solution was ultrafiltered with a membrane of 10 kDa cutoff (Millipore, Amicon) to remove unconjugated PEG chains. This resulted in spontaneous self-assembly of BDNF-PAMAM-PEG complexes that we will further refer to as “PEGylated PAMAM-based nanoparticles”.

The particle size, zeta potential, and polydispersity index (PDI) of BDNF, PAMAM 6, PAMAM 5.5, PAMAM-based nanoparticles, PEGylated PAMAM-based nanoparticles were determined with the Zetasizer Nano ZS apparatus (Malvern Instruments, Malvern, UK) equipped with a laser of 633 nm wavelengths. Data analysis was performed in automatic mode at 25 °C. Measured size was presented as the average value of 20 runs, with triplicate measurements within each run. Particle size distributions were obtained from measured diffusion coefficients.

The size distribution of PAMAM 6, PAMAM 5.5, PAMAM-based nanoparticles, PEGylated PAMAM-based nanoparticles was determined at mica surface by AFM (atomic force microscopy) technique. The nanoparticles were left to deposit on mica sheets (Continental Trade, Poland) placed in the diffusion cell over a 5 min, and then substrate was removed and rinsed for half an hour in ultrapure water. The samples were left for air-drying until the next day. Next, the dry sample was placed under a 7–10 nm AFM tip. The AFM measurements were carried out under ambient air conditions using the NanoWizard AFM (JPK Instruments AG, Berlin, Germany). The intermittent contact mode images were obtained in the air, using ultrasharp silicon cantilevers (NSC35/AlBS, MicroMash, Spain), and the cone angle of the tip was less than 20°. The images were recorded at the scan rate of 1 Hz for the six randomly chosen places. The images were flattened using an algorithm provided with the instrument. We captured all images in random areas within the scan size of 0.5 × 0.5 or 1 × 1 µm. BDNF, PAMAM 5.5, PAMAM6, PAMAM-based nanoparticles, and PEGylated PAMAM-based nanoparticles surface dimensions were determined using ImageJ software by gathering the number and coordinates of single protein/nanoparticles molecules.

### BDNF adsorption studies in PBS

Protein loading profile at PAMAM-based nanoparticles and PEGylated PAMAM-based nanoparticles with negative charge core as well as positive charge core were conducted by solution depletion ELISA technique (DY992, DY990, DY994, DY999, DY995, WA126, DY006, DY268, R&D Systems). Afterward, to determine unbound BDNF molecules more accurately after adsorption at PAMAM particles, a two-step Laser Doppler Velocimetry (LDV) technique with the aforementioned Malvern device's aid was used. LDV method exploiting the calibrating measurements uses various colloid particles (in our study modeled latex microparticles) for efficient monitoring of desorbed protein molecule concentration to determine maximum protein coverage at PAMAM nanoparticles.

### BDNF release studies in PBS

The protein release profile from PAMAM-based nanoparticles, PEGylated PAMAM-based nanoparticles with negative charge core as well as positive charge core was assessed by using ultrafiltration method with a 30 kDa cutoff membrane (Millipore, Billerica, MA, USA) in PBS at pH 7.4 and 0.15 M ionic strength. It was done in a two-stage procedure, where the first BDNF adsorption process was carried out for 1 h. According to the manufacturer’s protocol, the BDNF molecules released from nanoparticles with various charge core were quantified with the ELISA immunoassay method. Initially, the residual (unbound) BDNF concentration in the filtrate was determined immediately after adsorption at PAMAM nanoparticles by applying the sandwich ELISA technique to monitor the maximum concentration of unbound BDNF in the supernatant suspensions. Thus, it was possible to precisely determine the concentration of non-adsorbed BDNF molecules at PAMAM as well as PEG-ylated PAMAM nanoparticle surface. These measurements were utilized for determining the maximum coverage of neurotrophin under protein bulk conditions (0.1 mgL^−1^). Afterward, the concentration of desorbed BDNF for 24 h was quantified with UV–VIS spectroscopy and calculated according to ELISA standard curve.

### Cell culture and differentiation

SH-SY5Y neuroblastoma cells (human, ECACC; Sigma Aldrich, St. Louis, MO, USA) were used in this study. SH-SY5Y cells were incubated in culture plates in a proliferation medium containing Ham’s F-12 Nutrient Mixture (Thermo Fisher, Waltham, MA, USA) and minimum essential medium (MEM) (Sigma Aldrich, St.Louis, MO, USA) mixed in ratio 1:1 and supplemented with streptomycin (100 µg/mL), penicillin (100 U/mL), L-glutamine (2 mM) and 15% heat-inactivated fetal bovine serum (FBS) at 37^◦^C in saturated humidity atmosphere containing 5% CO_2_. The proliferation medium was changed every 2–3 days, and the cells were passaged when they reached 80% confluence. After the proliferation step, the cells were transferred into new culture plates, and incubated for 24 h with MEM supplemented with penicillin (100 U/mL), streptomycin (100 µg/mL), L-glutamine (2 mM) and 1% FBS. On the next day, the medium was changed to a differentiation medium consisting of MEM supplemented with penicillin (100 U/mL), streptomycin (100 µg/mL), L-glutamine (2 mM), 1% FBS, and Retinoic Acid (0.01 µmol/mL) (RA, Sigma Aldrich, St.Louis, MO, USA). The differentiation was carried out for 5 days, and the medium was changed every 2 days.

### 6-OHDA and nanoparticles cytotoxicity

Differentiated SH-SY5Y cells were incubated at a density of 3 × 10^4^ cells/well in 96-well plates for 24 h with MEM (without FBS) containing various concentrations of 6-hydroxydopamine (20-35 µmol/L) (6-OHDA, Sigma Aldrich, St.Louis, MO, USA). 6-OHDA was freshly prepared for each experiment to avoid oxidation. 6-OHDA remains the most widely used neurotoxin in Parkinson’s disease (PD) in vitro models [23], due to its structural similarity to dopamine (DA) and high affinity for the DA transporter, which enable it to destroy dopaminergic neurons selectively [24]. Therefore, for our study we chose to treat human neuroblastoma cell line SH-SY5Y with 6-OHDA, as it has been extensively described in the literature as a proper in vitro model for PD [25, 26]. Cytotoxicity of 6-OHDA was evaluated by exposing cells to different concentrations of this neurotoxin for 24 h at 37 °C, and cell viability was estimated by measuring toxicity using the MTT assay (Abcam, Cambridge, UK), which is based on the conversion of water-soluble 3-(4,5-dimethylthiazol-2-yl)-2,5-diphenyltetrazolium bromide to an insoluble formazan product, which has a purple color. At the end of the treatments, the medium was discarded, and a new medium containing 0.5 mg/ml was added. Cells were incubated with 50 μL of MTT reagent mixed with 50 µl MEM for 3 h, then 150 µl of the detergent solution was added to solubilize the colored crystals. Finally, absorbance was measured at 590 nm using Varioskan LUX Multimode Microplate Reader (Thermo Fisher, Waltham, MA, USA). Toxicity was calculated from the equation provided in the manufacturer's protocol.

After choosing a specific 6-OHDA concentration, the cells were incubated for 24 h with MEM (without FBS) containing 20 µmol/L of 6-hydroxydopamine. At the end of the treatments, the medium was discarded, and the following solution was added to each well: 20 µl of PAMAM-based nanoparticles or PEGylated PAMAM-based nanoparticles with different charged core in PBS (described above in "Preparation and characterization of PAMAM-based nanoparticles with negative and positive core" section) and 80 µl MEM without FBS. The last step was to assess the toxicity of various nanoparticles on differentiated SH-SY5Y cells using the MTT assay.

### BDNF release studied in neuroblastoma cell culture exposed to 6-OHDA

The concentration of released BDNF molecules was determined by exposing differentiated human neuroblastoma cells SH-SY5Y to 6-hydroxydopamine (6-OHDA) and nanoparticles with differently charged PAMAM core for 24 h at 37 °C. At the end of the treatments, the cell supernatant was discarded and collected to quantify BDNF concentration using UV–VIS spectroscopy calculated according to ELISA standard curve. Simultaneously we determined BDNF concentration in differentiated neuroblastoma cell lysate exposed to 6-OHDA what is crucial to enhancing nanoparticle efficacy. To obtain neuroblastoma cells exposed to 20 µmol/L, 6-OHDA lysates 3 × 10^4^ SH-SY5Y cells were trypsinized and washed in medium twice and then followed by four freezes (liquid nitrogen) and thaw (37 °C water bath) cycles. Large particles were removed by centrifugation (2,000 g for 10 min, followed by 13,000 g for 60 min at 4 °C), then lysate was filtered through a 0.22 μm mesh, and aliquots were stored at – 80 °C. The protein content was determined by the ELISA assay.

### PAMAM-based nanoparticles and PEGylated PAMAM-based nanoparticles behavior in cell culture

In addition, we further investigated the behavior of our nanoparticles loaded with BDNF in SH-5YSY cell culture by determining of green fluorescence of PAMAM-AF488 conjugates. Differentiated SH-SY5Y cells and previously treated with 6-OHDA (protocol described in "6-OHDA and nanoparticles cytotoxicity" section) were incubated at a density of 3 × 10^4^ cells/well in 96-well plates with BDNF-PAMAM-AF488 and BDNF-PAMAM-AF488-PEG nanoparticles (0.1 μg/mL protein loading) for 24 h. After the labeled AF488 nanoparticles were removed and the wells were washed twice with PBS, the cells were subjected to examinations by spectrofluorimetry evaluation using Varioskan LUX Multimode Microplate Reader.

### Flow cytometry

To determine a mean fluorescence intensity (MFI) of nanoparticles PAMAM 5.5 and PAMAM 6 conjugated with fluorochrome AF488, the differentiated and treated with 6-OHDA cells SH-SY5Y (protocol described in "6-OHDA and nanoparticles cytotoxicity" section) were incubated at a density of 3 × 10^4^ cells/well with PAMAM 5.5-AF488, PAMAM 5.5-AF488-BDNF(0.1 μg/mL), PAMAM 5.5-AF488-BDNF(0.1 μg/mL)-PEG, PAMAM 6-AF488, PAMAM 6-AF488–BDNF(0.1 μg/mL) and PAMAM 6-AF488–BDNF(0.1 μg/mL)-PEG each in 10 separate repetitions for 24 h. The cells were then washed with PBS, incubated for 3 min with trypsin, transferred to a cytometric tube, washed twice in PBS, and resuspended in 200 µl PBS. Fluorescence was measured and the data were analyzed using a LSRII flow cytometer (BD Biosciences) and the BD FACSDiva software. 10 000 events were acquired to determine the MFI.

### Detection of lipid peroxidation

Malondialdehyde (MDA) is the most abundant and stable individual indicator of the level of the aldehydic products that can be produced from the free radical attack on polysaturated fatty acids indicating lipid peroxidation present in cell cultures. The cells were incubated for 24 h with different concentrations of BDNF, BDNF-PAMAM dendrimer nanoparticles, or PEG-ylated BDNF-PAMAM dendrimer nanoparticles, adding 20 µl to each well of a selected solution prepared in PBS (described in "Preparation and characterization of PAMAM-based nanoparticles with negative and positive core" section) and 80 µl MEM without FBS. For this purpose, after the completion of 24 h of incubation with various types of nanoparticles, the cell supernatant was collected from each well (3 × 10^4^ cells/well) for MDA analysis.

The MDA analysis in cell supernatant was determined using reverse-phase, high-performance liquid chromatography (HPLC)-spectrophotometric method according to Ref. [27]. HPLC (varian vista series) was performed on an Agilent Zorbax column (300A). Qualitative and quantitative analyses of MDA release by the differentiated human neuroblastoma cells SH-SY5Y treated 6-OHDA were performed by integrating the retention times and the peak areas compared with known concentrations of MDA standard prepared in the same solvent. 1,1,3,3-tetraethoxypropane (TEP) standards were freshly prepared daily. The solvent blank was absolute ethanol (400 mL/L) in distilled water. The stock standards were 10 and 100 mM TEP, and the working calibrants were 0.0, 0.25, 0.50, 0.75, and 1.0 mM TEP, prepared from dilution of the 100 mM TEP with absolute ethanol (400 mL/L). The HPLC calibration was performed for each run. Samples were calculated as mM MDA equivalent from the TEP standard calibration (1:1 conversion under acidic conditions).

### Detection of mitochondrial membrane potential

JC-1 (Caymanchem, MI, USA) is a lipophilic, cationic dye that can selectively enter mitochondria. In healthy cells, JC-1 forms aggregates, that display strong fluorescence intensity with excitation and emission at 535 and 595 nm. In apoptotic/unhealthy cells, JC-1 exists as monomers that show strong fluorescence intensity with excitation at 485 nm and emission at 535 nm. Differentiated and treated with 6-OHDA cells SH-SY5Y (protocol described in "6-OHDA and nanoparticles cytotoxicity" section) were incubated at a density of 3 × 10^4^ cells/well with PAMAM 5.5, PAMAM 6, PAMAM 5.5-BDNF(0.02 μg/mL), PAMAM 6–BDNF(0.02 μg/mL), PAMAM 5.5–BDNF(0.02 μg/mL)-PEG, PAMAM 6-BDNF(0.02 μg/mL)-PEG, PAMAM 5.5-BDNF(0.1 μg/mL), PAMAM 6-BDNF(0.1 μg/mL), PAMAM 5.5-BDNF(0.1 μg/mL)-PEG, PAMAM 6-BDNF(0.1 μg/mL)-PEG, PAMAM 5.5-PEG, PAMAM 6-PEG, each in 16 separate repetitions for 24 h. The cells were then incubated with JC-1 Staining Solution for 15 min in a CO_2_ incubator at 37 °C according to the manufacturer's protocol. Fluorescence was measured with Ex535nm/Em595nm and Ex485nm/Em535nm using a Varioskan Lux Reader (Thermo Fisher Scientific, MA, USA). The ratio of fluorescence intensity of aggregates to the fluorescence intensity of monomers was used as an indicator of cell health.

### Confocal microscopy

To determine the morphological changes, the SH-SY5Y cells were seeded at a density of 1 × 10^5^ cells/well in a 4-well chamber slide, differentiated with RA, and treated with 6-OHDA. Next, the cells were incubated with: PAMAM 5.5, PAMAM 5.5-PEG, PAMAM 5.5-BDNF (0.1 µg/mL), PAMAM 5.5-BDNF (0.1 µg/mL)-PEG, PAMAM 6, PAMAM 6-PEG, PAMAM 6-BDNF (0.1 µg/mL) and PAMAM 6-BDNF (0.1 µg/mL)-PEG for 24 h. Subsequently, the cells were fixed with 70% ethanol for 15 min and washed twice with PBS. For cell membrane staining the slides were incubated with 1:100 wheat germ agglutinin-Texas Red X (ThermoFisher, Waltham, MA, USA) in HBSS buffer for 20 min. After washing, the slides were counterstained with DAPI, mounted, and examined using Carl Zeiss LSM 700 microscope (Zeiss, Jena, Germany). The cell nuclei dimensions were measured at their widest point using ZEN software. For each specimen, at least 200 measurements were taken. For cellular localization of dendrimers, the RA and 6-OHDA treated SH-SY5Y cells were incubated with Alexa Fluor 488 labeled: PAMAM 5.5, PAMAM 5.5-PEG, PAMAM 6, and PAMAM 6-PEG for 24 h. Subsequently, the cells were fixed with 70% ethanol for 15 min, washed twice with PBS, and counterstained with DAPI. The slides were examined using Carl Zeiss LSM 700 microscope (Zeiss, Jena, Germany).

### Statistical analysis

All presented data are expressed as means ± standard deviation (SD) from at least three independent experiments. Statistical analysis among each study group was performed using Kruskal–Wallis test. Two-Way ANOVA was used for analysis between experimental groups. *p* < 0.05 was considered statistically significant.

## Results

### Characterization of PAMAM- and PEGylated PAMAM-based nanoparticles with differently charged PAMAM cores

**Fig. 1:**
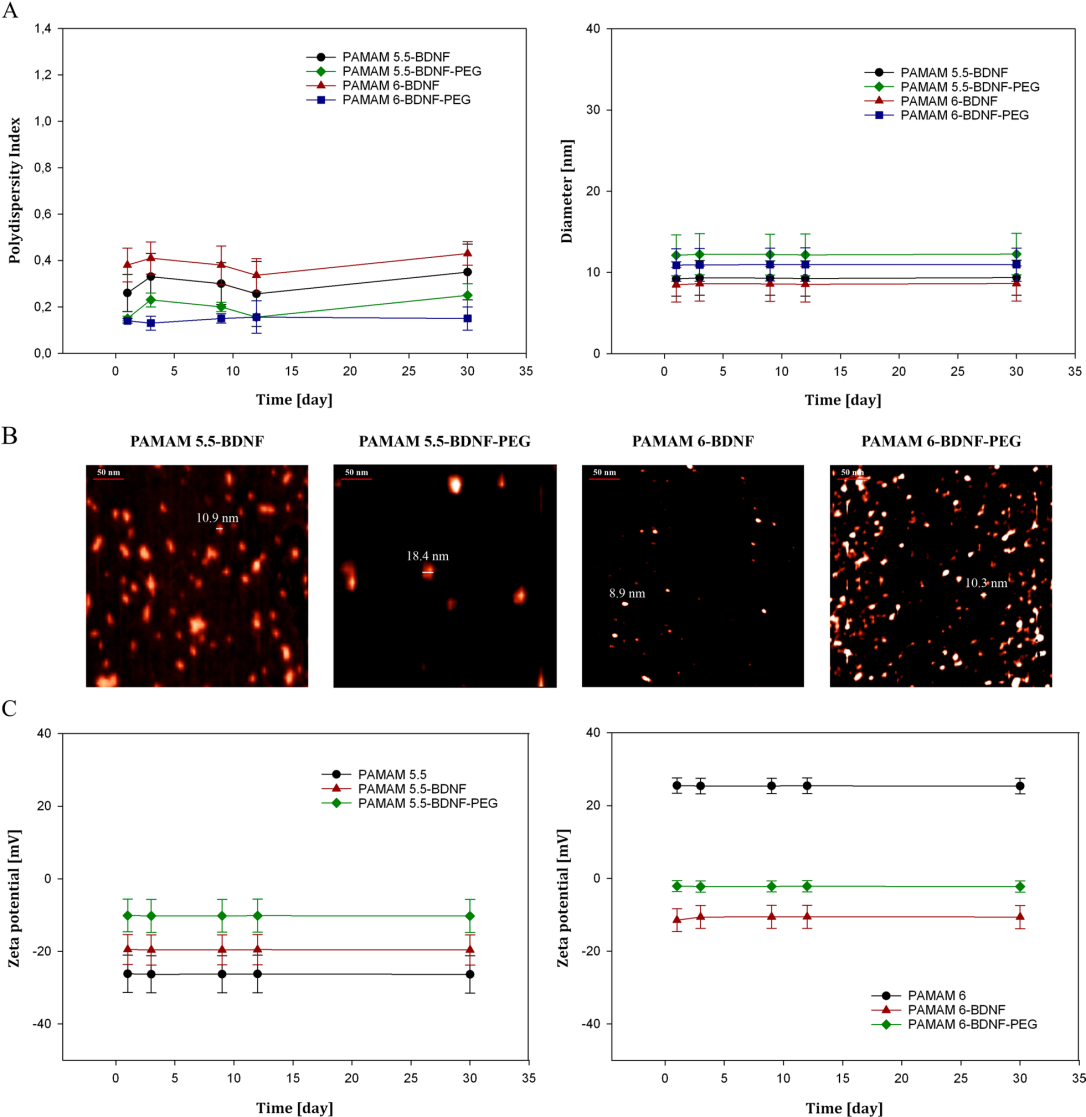
Physicochemical characterization of BDNF-PAMAM as well as PEG-ylated BDNF-PAMAM nanoparticles with positively and negatively charged dendrimers core. Serum stability of nanoparticles studied using DLS. Particle size distribution (**A**) and hydrodynamic diameter (**B**) of BDNF-PAMAM5.5, BDNF-PAMAM5.5-PEG, BDNF-PAMAM6, BDNF-PAMAM6-PEG nanoparticles, measured against PAMAM dendrimers core nanoparticles (without BDNF). All samples were incubated in PBS with 10% FBS over 30 days. All experiments were performed in triplicates and error bars represent mean ± standard deviation (SD) (**A** and **C**). The dependence of the zeta potential of positively charged PAMAM 6 or negatively charged PAMAM5.5 dendrimers core on the initial BDNF concentration of 0.1 mgL^−1^ in the suspension in PBS, pH 7.4, 0.15 M ionic strength (**C**). Representative AFM “high” diagrams (**B**) of BDNF-PAMAM5.5, BDNF-PAMAM5.5-PEG, BDNF-PAMAM6, BDNF-PAMAM6-PEG nanoparticles adsorbed at mica surface at 0.15 M pH 7.4 in PBS at a scan area of 0.5 × 0.5 µm and structure of PAMAM-based nanoparticles after cross-section

To understand the BDNF release mechanism, we examined the physical characteristics of this encapsulation-free release system—based on particle size, polydispersity index (PDI), zeta potential, drug loading (DL)%, and encapsulation efficiency (EE)%. PAMAM-based nanoparticle size was characterized by atomic force microscopy (AFM) and dynamic light scattering (DLS). DLS studies indicated that all nanoparticle preparations were under 13 nm in hydrodynamic diameter and had an optimal polydispersity index of less than 0.4. PAMAM-based nanoparticles with negatively and positively charged core were spherical, with an average diameter of 9 ± 1 nm and a polydispersity index of 0.4 ± 0.15 measured by DLS (Fig. 1A). DLS analysis for PEGylated PAMAM-based nanoparticles with negatively and positively charged core revealed a mean diameter of 12 ± 1.1 nm, with a relatively low PDI of less than 0.22. As shown in Fig. 1A, the mean hydrodynamic diameter value obtained for BDNF-PAMAM nanoparticles was significantly lower from the PEG-ylated ones as opposed to the PDI value showing that PEG-functionalization influences the average size and polydispersity of the nanoparticles. The PDI and hydrodynamic diameter of the nanoparticles over time are shown in Fig. 1A and C respectively. All PAMAM-based nanoparticles with negatively and positively charged core showed size stability in the presence of serum. Fluctuations in nanoparticle hydrodynamic diameter were within the range of 15 nm in all sample groups at the end of 30 days. The PDI of PEG-ylated groups was also maintained at less than 0.2, indicating that they remained in the monodisperse state without forming aggregates over the same time. As protein/drug delivery systems, all tested PAMAM-based nanoparticles remained stable at the high salt concentration for over 30 days. Therefore, this spontaneous self-assembly nanocomplex can be used as an injectable pharmaceutical agent without sonication or other extensive agitation.

Using AFM, we could visualize an average nanoparticle diameter on the mica surface and the size range of nanoparticles adsorbed under the diffusion-controlled transport condition at pH 7.4 and ionic strength 0.15 M. AFM (Fig. 1B) suggested that PAMAM-BDNF-PEG nanoparticle was around 9 ± 2 nm in diameter and was smaller than PAMAM-BDNF formed nanoparticles with a diameter of 11 ± 2 nm. In addition, AFM studies revealed that all tested nanoparticles were spherical and distributed on the mica surface uniformly without adhesion or aggregation. This enabled us to precisely determine their sizes by cutting down the possibility of tip convolution artifacts. Afterwards, the prepared BDNF-PAMAM as well as PEG-ylated BDNF-PAMAM nanoparticles were physicochemically characterized in terms of electrophoretic mobility/zeta potential in PBS buffer without calcium and magnesium ions. The BDNF adsorption on positively and negatively charged PAMAM dendrimers core at 7.4 pH and 0.15 M ionic strength proceeds according to the bulk diffusion control transport mechanism. The experimental data showed surface charge stability of PAMAM core, BDNF-PAMAM and PEG-ylated BDNF-PAMAM nanoparticles obtained in the primary adsorption experiments (shown in Fig. 1C) with initial concentration of BDNF 0.1 mgL^−1^ in the suspension (after mixing). Zeta potential measurements showed that the core of the PAMAM 5.5 was negatively charged (− 26  ± 5.2 mV), increasing with BDNF adsorption up to -19  ± 4.2 mV. Furthermore, PEG adsorption at BDNF-PAMAM nanoparticles increased zeta potential value even more (to -10  ± 4.5 mV), probably because of a reduction in the number of charged groups on the nanoparticle surface if they are, in part, utilized as anchoring points for PEG binding or due to chelation of cations by the PEG chains. On the other hand, the zeta potential of PAMAM 6 was positive (25  ± 2.1 mV), decreasing up to − 11  ± 3.2 mV after adsorption of slightly positively charged BDNF molecules (5  ± 2.5 mV). The observed significant adsorption of a positively charged molecule on a positively charged surface at above mentioned bulk conditions is anomalous from the mean-field Gouy-Chapman theory. As discussed in Refs. [28, 29] this can be explained by highly heterogeneous charge distribution over the BDNF molecule or conformational changes in the protein structure. It can be noted that for 0.1 mgL^−1^ BDNF concentration, the change in the zeta potential of PAMAM 6 dendrimer (positively charged core) is significant, whereas, in case of PAMAM 5.5 dendrimer (negatively charged core), there is just a slight change in the zeta potential. Irrespective of the initial dendrimer core charge, PEG-ylation of the PAMAM-BDNF surface significantly increases the zeta potential of the nanoparticle. In the case of PAMAM 6, PEG-ylation of the PAMAM-BDNF nanoparticles neutralizes the initial charge of the particle, as the zeta potential approaches the value close to − 2.5 ± 1.5 mV.

### BDNF loading and releasing from PAMAM- and PEGylated PAMAM-based nanoparticles with differently charged PAMAM cores in PBS buffer

To determine the possibility of desorption from PAMAM- as well as PEGylated PAMAM-based nanoparticles with differently charged PAMAM core, once adsorption reached equilibrium and before the in vitro release profile, was performed, we studied the BDNF release in PBS buffer.

**Fig. 2:**
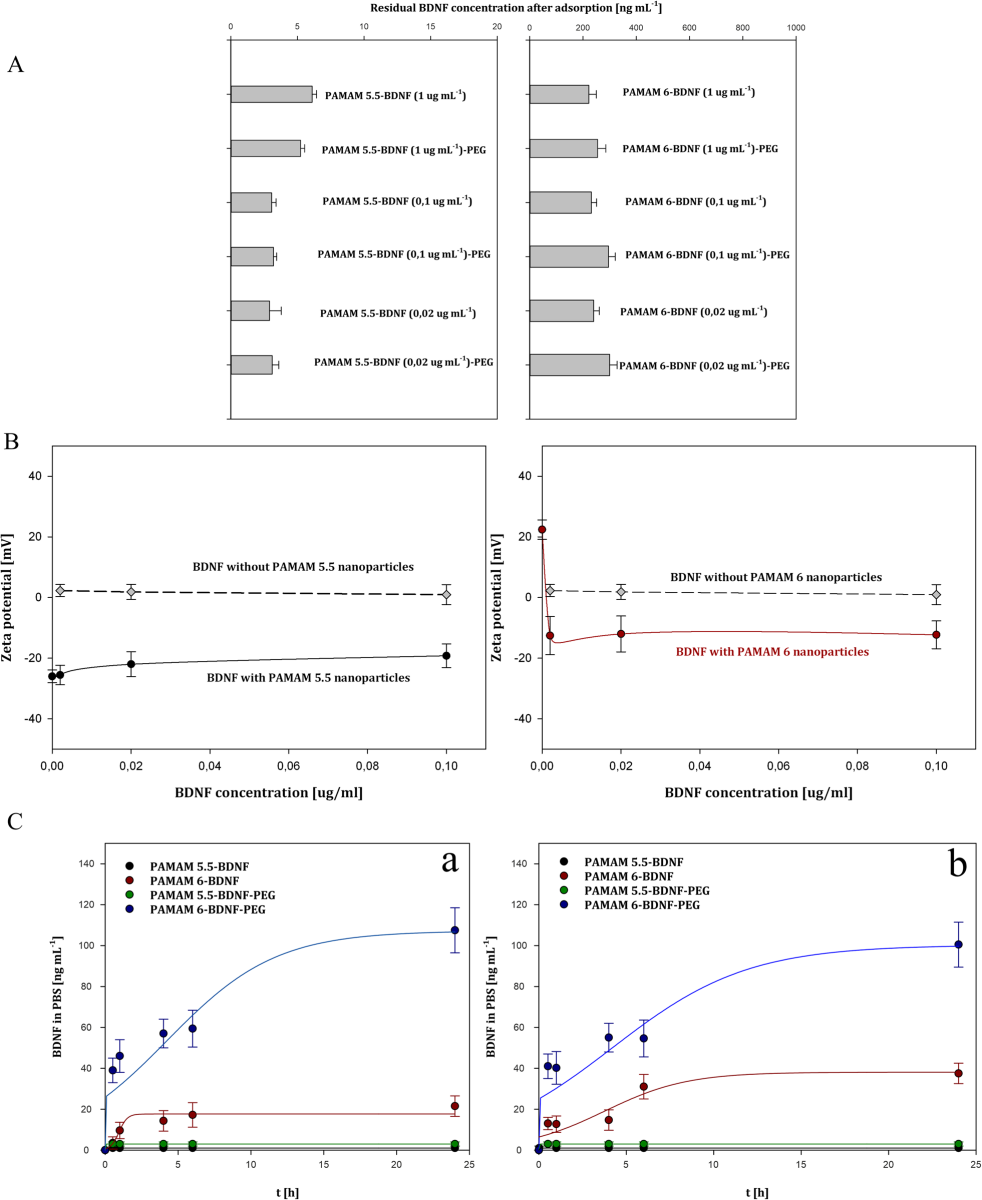
Loading and releasing of BDNF from BDNF-PAMAM as well as PEG-ylated BDNF-PAMAM nanoparticles with positively and negatively charged dendrimers core. Encapsulation efficiency **A**: Dependence of residual BDNF concentration after adsorption step at PAMAM nanoparticles on initial protein concentration after 1 h’s adsorption. Encapsulation efficiency **B**: The dependence of the zeta potential of positively charged PAMAM 6 or negatively PAMAM5.5 dendrimers core on the initial BDNF concentration in the suspension 0.1 mgL^−1^ in PBS after mixing pH 7.4, 0.15 M ionic strength. **C** BDNF release profile from BDNF-PAMAM and PEG-ylated BDNF-PAMAM nanoparticles with various charge dendrimer core and concentrations 0.1 mgL^−1^ (**a**) and 1 mgL^−1^ (**b**) in PBS

Initially, adsorption of slightly positively charged BDNF molecules to negatively as well as positively charged PAMAM dendrimers core surfaces was precisely determined to control the concentration of unbounded protein molecule and protein-laden nanoparticles structure. For every tested system, the saturation concentration of the protein had to be determined empirically. PAMAM nanoparticle was characterized with significant changes in its apparent zeta potential during adsorption, which we efficiently monitored using DLS method. After BDNF loading into PAMAM-based nanoparticles, we determined the dependence of the zeta potential of nanoparticles on the initial concentration of BDNF in the PAMAM suspension (after mixing). As is depicted in Fig. 2B (left side), for adsorption at PAMAM 5.5, the PAMAM 5.5-BDNF zeta potential slightly increases with increasing BDNF concentration and approaches the value of − 19 mV, which is considerably below the zeta potential of BDNF molecules determined in bulk (5 mV at 0.15 M ionic strength). For the range of tested BDNF concentrations (0.1–100 mg L^−1^), the zeta potential of the BDNF is relatively stable. Importantly, at pH 7.4, BDNF molecules carry a net positive charge and adsorb onto a negatively charged uniform surface according to the mean-field theory because both BDNF and PAMAM core exhibit opposite signs of zeta potentials. Moreover, the electrophoretic mobility of BDNF-PAMAM 5.5 core dendrimers is far from the value obtained for the electrophoretic mobility of BDNF in bulk, and it corresponds to the formation of unsaturated BDNF structures on PAMAM cores. As is depicted in Fig. 2B (right side), the zeta potential of PAMAM 6 dendrimers abruptly decreases with BDNF adsorption. It is quite unexpected as BDNF zeta potential was equal to + 5 mV, and the PAMAM 6 surface was also strongly positive, having a zeta potential of + 25 mV. According to our knowledge, our study for the first time shows such an anomalous BDNF-PAMAM 6 interaction. However, similar behavior of different proteins deposition under physiological conditions have been previously observed by Wasilewska et al. [29], Malmsten [30], Ortega-Vinuesa et al. [31] Toscano and Santore [32], Kalasin and Santore [33], and others. Overall, the experimental results are shown in Fig. 2 B suggest that an electrostatically driven adsorption of BDNF at PAMAM can be expected for both positively and negatively charged dendrimer cores and slightly positively charge BDNF molecules. This hypothesis was checked experimentally, as described below.

We used ELISA to determine residual (free) BDNF concentration after adsorption at PAMAM core dendrimers with opposite surface charge as well as after PEGylation of PAMAM-BDNF nanoparticles (Fig. 2A). As can be observed, for every tested initial BDNF concentration (from 0.02 to 1 mg L^−1^), the concentration of unbounded protein molecules in PBS was negligible only for positively charged dendrimer molecule (PAMAM 5.5), indicating that adsorption onto 10 mg L-1 of PAMAM nanoparticles was almost complete only for oppositely charged molecules. The concentration of unbounded BDNF protein was significantly higher in BDNF-PAMAM 5.5 than in BDNF-PAMAM 6 nanoparticles, which correlates with the dendrimer charge. As discussed elsewhere [34], by knowing the electrophoretic mobility, one can calculate the average number of free charges per protein molecule expressed in coulombs from the Lorenz − Stokes relationship under physiological conditions. The value of the uncompensated charge per BDNF molecule reaches 0.9e (the BDNF molecule acquired a net positive charge which is around three times smaller than for HSA). Under these conditions, double-layer forces were remarkably weak due to decreasing protein net charge, which indeed enhances the possibility of BDNF aggregation for this pH range in accordance with the hydrodynamic diameter measurements of native BDNF (not shown). According to Jiang et al. [35], BDNF forms large and highly heterogeneous aggregates with an effective diameter of 600 nm and PDI greater than 0.4. Furthermore, thus in this form, it cannot be used as an injectable drug agent. Our approach resolves this problem by mixing BDNF and PAMAM in appropriate concentrations and using simple electrostatic interactions between BDNF molecules and PAMAM surfaces which indeed hampers the possibility of BDNF aggregation, in accordance with the aforementioned measurements of the hydrodynamic diameter (Fig. 1B). In the case of PAMAM 5.5 nanoparticles, the residual BDNF concentration after adsorption is remarkably lower than in PAMAM 6 nanoparticles, suggesting that after the adsorption at negatively charged dendrimer core, the remaining scarce unbound BDNF molecules are negligible.

For both types of nanoparticle concentrations (0.1 and 1 mgL^−1^), the first phase in releasing profile of BDNF is characterized by a fast release of protein molecules from positively charged dendrimer surfaces, which probably results from weak electrostatic interactions between protein adjoining dendrimer surface. In the case of PEGylated PAMAM 6 core nanoparticles, for both BDNF loaded concentrations, spontaneous electrostatic interaction led to the release of less than 100 ng mL^−1^ of BDNF for 24 h and gave approximately 10% of loaded protein. On the other hand, PAMAM 6 core nanoparticles without a PEG layer release five times less protein than PEG-ylated ones.

### Nanoparticle’s toxicity

In order to determine the possibility of toxicity of PAMAM-based nanoparticles with differently charged PAMAM core in biomedical applications, we examined the viability of differentiated neuroblastoma SH-SY5Y cells previously exposed to 6-OHDA and then administered nanoparticles. First and foremost, we differentiated SH-SY5Y cells by combining RA treatment and lowering FBS concentration in cell culture according to our previous report [36].

**Fig. 3:**
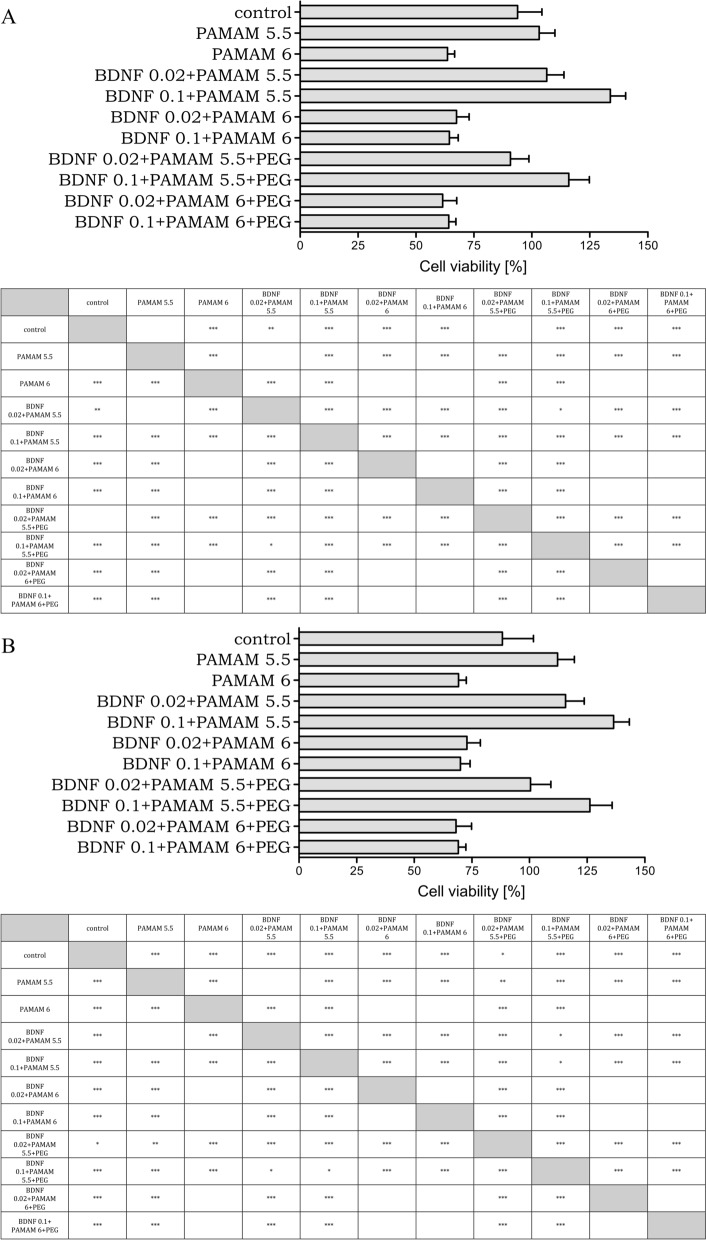
Cell viability of differentiated human neuroblastoma cell line SH-SY5Y exposed to 20 µmol/L 6-OHDA neurotoxin and treated with differently charged PAMAM cores, PAMAM- and PEGylated PAMAM-based nanoparticles. The control (100% viability) are cells treated 7 h (part A) and 24 h (part B) with 20 µmol/L 6-OHDA only. The data represent means ± SD for 30 experiments. Data distribution was tested using Shapiro–Wilk test. To compare two analysed datasets the unpaired t-test was used. The analysis of variances between different time-points was performed using one-way ANOVA. *—p < 0.05, **—p < 0.01, ***—p < 0.001

Then, to establish an experimental dosage of 6-OHDA for testing the toxicity of PAMAM- and PEGylated PAMAM-based nanoparticles with differently charged PAMAM cores, we examined differentiated SH-SY5Y cells’ responsiveness to various concentrations of 6-OHDA for 24 h using two complementary methods: MTT assay and flow cytometry (Supplementary materials). The reduction of cell viability was employed here as an indicator of cell proliferation and nanoparticle toxicity. We established that exposure to 20 µmol/L 6-OHDA resulted in a significant 6 and 12% decline in cell viability after 7 h and 24 h of incubation. For further studies, we chose this dose of 6-OHDA to determine the cytotoxicity of PAMAM, PAMAM- and PEGylated PAMAM-based nanoparticles with differently charged PAMAM cores on differentiated SH-SY5Y cells after 7 and 24 h of incubation (Fig. 3).

As we observed, the nanoparticles with negatively charged PAMAM core (5.5 generation) lead to higher cell viability of differentiated human neuroblastoma cell line SH-SY5Y treated with the 6-OHDA neurotoxin in vitro for both incubation time. In addition, the MTT assay demonstrated that 10 mgL^−1^ of PAMAM 5.5 dendrimers significantly increases cell viability, whereas positively charged PAMAM 6 dendrimers significantly decrease cell viability compared to control (Supplementary Materials). The data presented in Fig. 3A further shows that 7 h incubation of neuroblastoma cells with PAMAM-BDNF nanoparticles with negatively charged core increases cell viability from 103% (initial value obtained for PAMAM 5.5 without BDNF) to 134% in the case of PAMAM 5.5-based nanoparticles with initial protein concentration equal to 0.1 mgL^−1^. After increasing incubation time up to 24 h, we noticed that cell viability from 112% (initial value obtained for PAMAM 5.5 without BDNF) to 137% in the case of PAMAM 5.5-based nanoparticles with initial protein concentration equal to 0.1 mgL^−1^. Moreover, after adsorption of 25 mgL^−1^ PEG at nanoparticles with negatively charged dendrimer core, a distinct decrease in metabolic cell activity was observed compared to PAMAM5.5 and PAMAM 5.5-BDNF nanoparticles for both protein concentration and incubation time. On the contrary, a similar decrease in cell viability was observed for positively charged PAMAM6, PAMAM6-BDNF, and PEGylated PAMAM6-BDNF compared to controls, suggesting that nanoparticles with positively charged core are less biocompatible with differentiated cells and presumably show non-specific toxicity. Thus, those measurements indicate that positively charged PAMAM core dendrimer markedly decreases in vitro cellular viability in the presence of neurotoxin.

### In vitro BDNF release profile from PAMAM- and PEGylated PAMAM-based nanoparticles with differently charged PAMAM cores

**Fig. 4:**
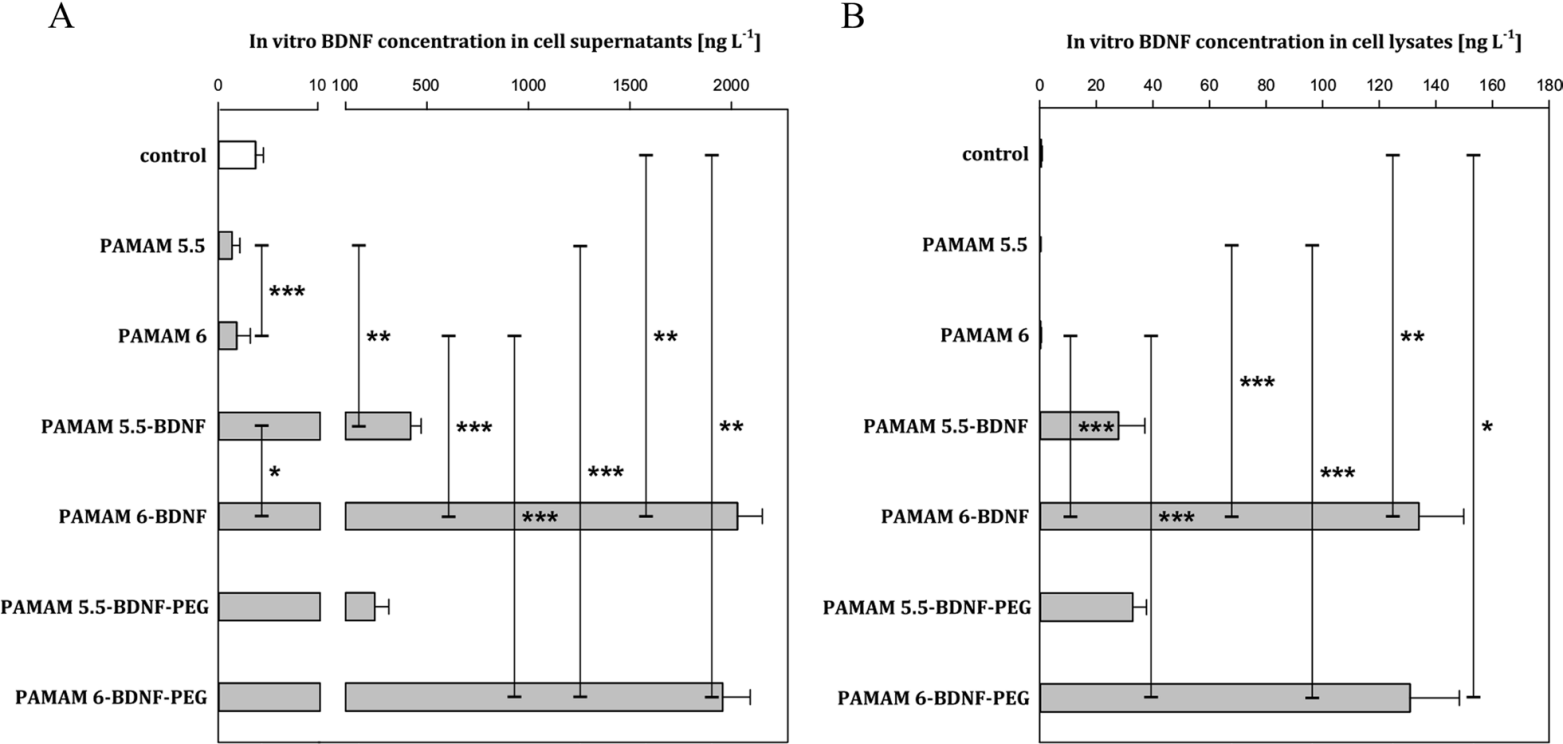
Desorption characteristic of BDNF from BDNF-PAMAM and PEG-ylated BDNF-PAMAM nanoparticles with differently charged dendrimer core in cell culture lysates and supernatant under carriers loading of protein concentrations equal to 0.1 mgL^−1^. BDNF detection by ELISA over 24 h in cells incubated with PAMAM 5.5 (no BDNF), PAMAM 5.5- BDNF, PAMAM 5.5- BDNF-PEG, PAMAM 6 (no BDNF), PAMAM6—BDNF, PAMAM 6- BDNF-PEG. The data represent means ± SD for five experiments. Data distribution was tested using Shapiro–Wilk test. To compare two analysed datasets the unpaired t-test was used. The analysis of variances between different time-points was performed using one-way ANOVA. *—p < 0.05, **—p < 0.01, ***—p < 0.001.In supernatants from control cells BDNF, was released at the concentration of 4 ngL^−1^. Interestingly, we observed that the application of positively as well as negatively charged dendrimers (6 and 5.5 generation, respectively) to the cell culture significantly decreased BDNF concentration in cells supernatant (Fig. 4). There was no detectable BDNF concentration in cell lysates, neither in controls nor after treating PAMAM 5.5 or PAMAM 6 dendrimers. We also observed that BDNF concentration in cell supernatant was significantly lower in the case of PAMAM5.5- compared to the PAMAM6-based nanoparticles

Next, we aimed to determine whether BDNF from dendrimer-based nanoparticles is sustainably released in the in vitro setting. Thus, we investigated the suitability of negatively and positively charged PAMAM dendrimers and PAMAM- and PEGylated PAMAM-based nanoparticles with differently charged PAMAM cores to effectively transport BDNF to differentiated neuroblastoma cells exposed to 6-OHDA. To assess the distribution of applied various dendrimer-neurotrophin conjugates, we analyzed BDNF concentrations in cell lysates as well as culture supernatant using ELISA after 24 h post-treating. The cells exposed to RA and 20 µmol/L 6-OHDA served as controls. The results are summarized in Fig. 4.

As shown in Fig. 4, PEG-ylation of nanoparticles, regardless of the dendrimer core charge, leads to a decrease in BDNF concentration in the supernatant, whereas there is no such result in cell lysates of PEG-ylation. For BDNF release profile in PBS from PEG-BDNF-PAMAM nanoparticles with PAMAM 6 dendrimer core after 24 h of treating, we observed significantly higher desorption of BDNF compared to nanoparticles without PEG dendrimer core (Fig. 2C) what is in opposition to in vitro releasing profile. On the other hand, in in vitro setting, we found that BDNF desorption from the nanoparticles based on negatively, and positively charged dendrimers core, is in good accordance with the aforementioned release profile presented in PBS (Fig. 4).

### Cellular uptake of PAMAM- and PEGylated PAMAM-based nanoparticles with differently charged PAMAM cores

**Fig. 5:**
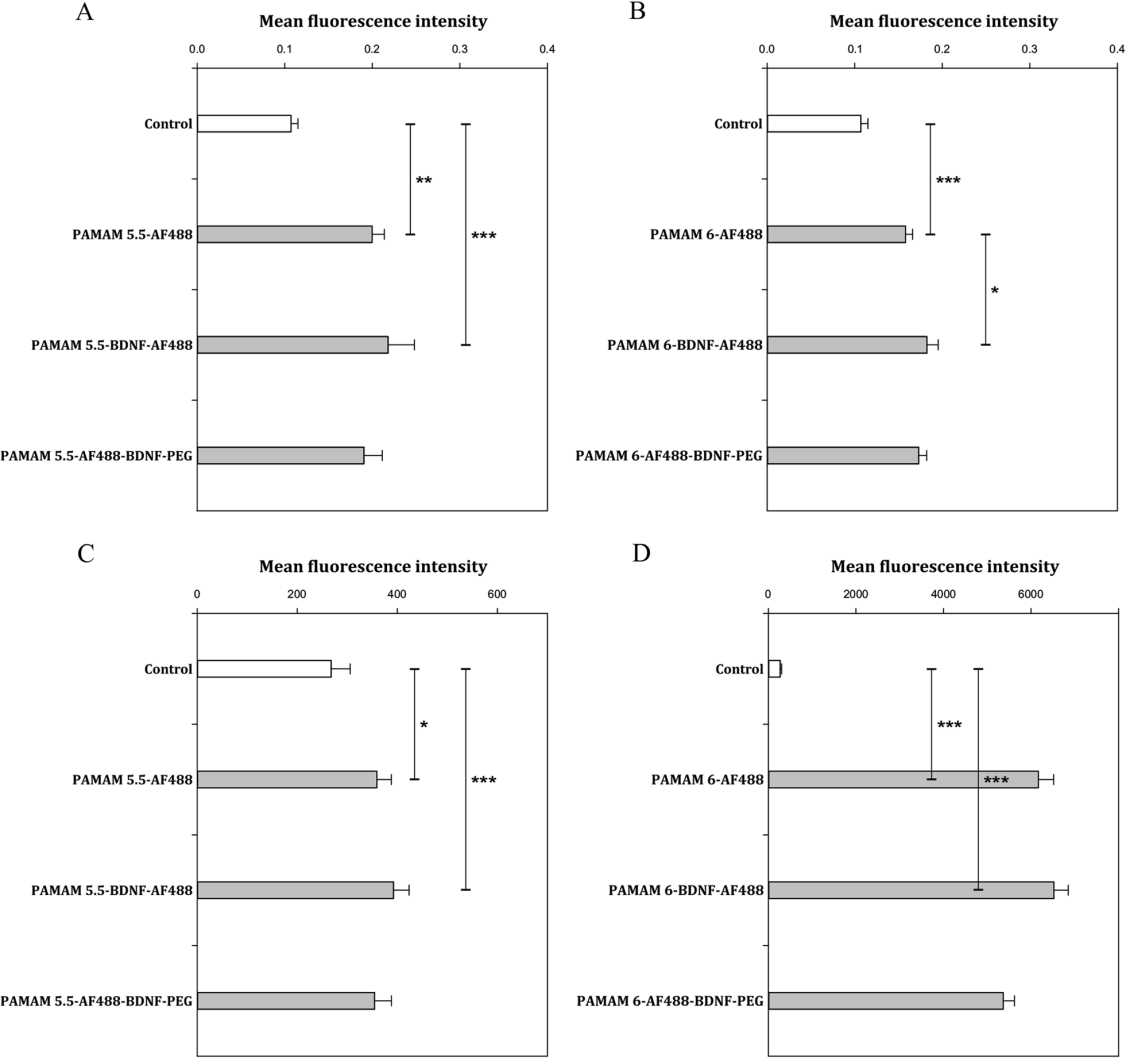
Immunofluorescence assessment (through spectrofluorimetry part A and B) and (through flow cytometry part C and D) of dendrimers-based nanoparticles localization in differentiated human neuroblastoma cell line SH-SY5Y upon treatment for 24 h. Data is presented as the mean ± SD (*n* = 12). Data distribution was tested using Shapiro–Wilk test. To compare two analysed datasets the unpaired t-test was used. The analysis of variances between different time-points was performed using one-way ANOVA. *—p < 0.05, **—p < 0.01, ***—p < 0.001

Cellular uptake of PAMAM 5.5-AF488 (no BDNF), PAMAM 5.5-AF488-BDNF, PAMAM 5.5-AF488-BDNF-PEG, PAMAM 6-AF488 (no BDNF), PAMAM6-AF488-BDNF-, PAMAM 6-AF488-BDNF-PEG in differentiated human neuroblastoma cell line SH-SY5Y treated with 6-OHDA was examined through spectrofluorimetry with a fixed concentration of BDNF (0.1 mg L^−1^). As shown in Fig. 5A and B, PAMAM dendrimers without protein, PAMAM- as well as PEGylated PAMAM-based nanoparticles with differently charged cores seem rather coated on SH-SY5Y cell surface than internalized by cells after 24 h of incubation. As shown in Fig. 5A and B, there was an observable change in green fluorescence intensity for PAMAM-AF488 dendrimers compared to control cells without carriers, suggesting that NPs can effectively be adsorbed on cells membrane. However, PAMAM5.5-AF488 and PAMAM6-AF488 show different fluorescence intensity due to the varying functional groups on their surfaces, and this results in differences in fluorescent labeling efficiency. Therefore, PAMAM 5.5 and PAMAM 6 cannot be directly compared in the case of cellular uptake. As can be seen in Fig. 5, PEG-ylation of nanoparticles results in lower green fluorescence intensity than for NP without PEG, such as PAMAM-BDNF and PAMAM alone for both dendrimer types.Due to the limitations of the spectrofluorimetry method, we were unable to assess whether nanoparticles have entered the cells or not unequivocally; thus, we used flow cytometry to investigate this matter further. Flow cytometry analysis was carried out better to understand the cellular uptake efficiency of the developed NPs. As seen in Fig. 5C and D, PAMAM-based nanoparticles harboring BDNF presented more pronounced green fluorescence than PAMAM dendrimers without BDNF.

In addition, we further investigated using confocal microscopy the cellular localization of PAMAM and PEG-PAMAM nanoparticles in the neural-like cellular environment previously exposed to the neurotoxin. Confocal analysis was conducted to assess the cellular uptake of Alexa Fluor 488 labeled PAMAM 5.5, PAMAM 5.5-PEG, PAMAM 6, and PAMAM 6-PEG nanoparticles qualitatively at 24 h.

**Fig. 6:**
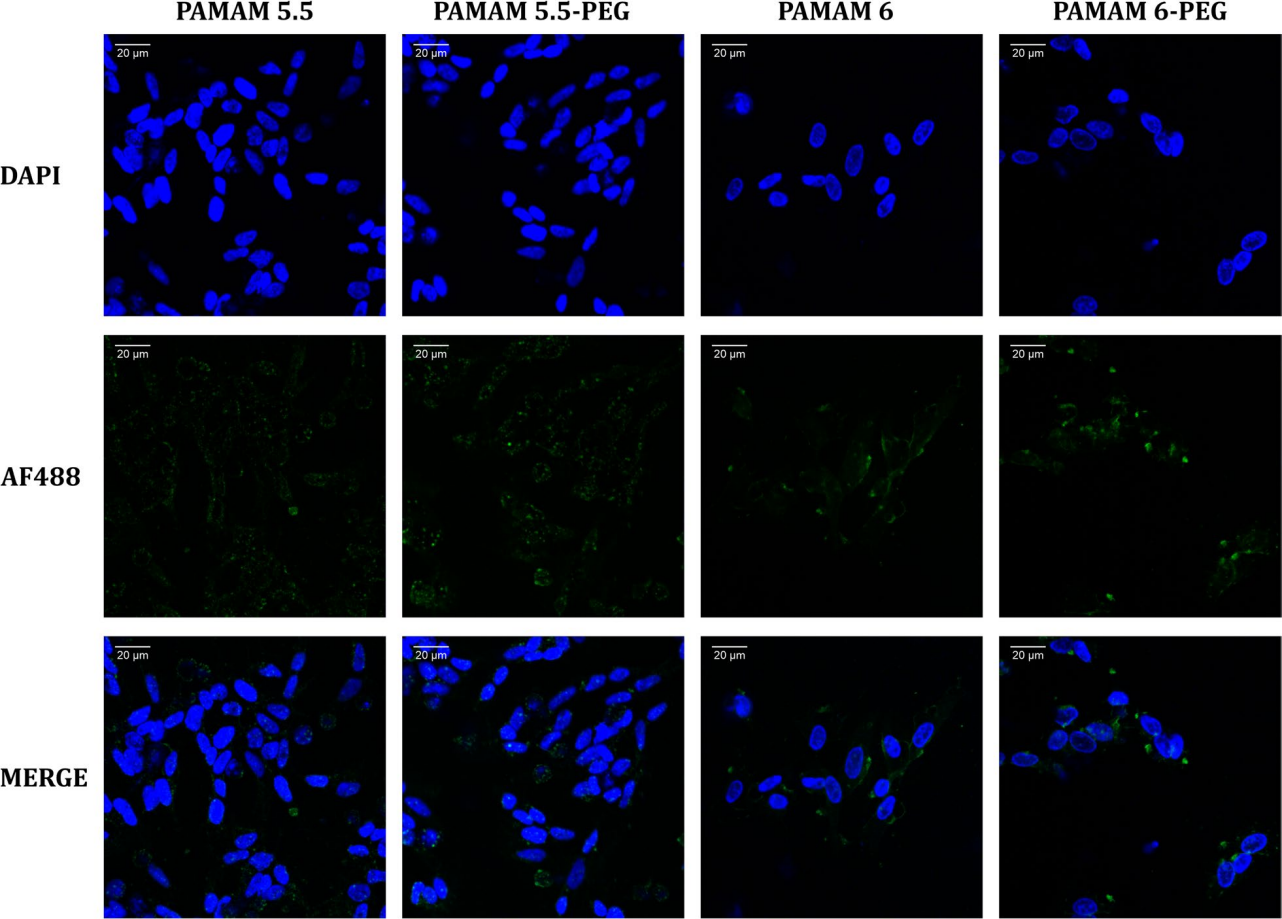
Cellular localization of Alexa Fluor 488 labeled PAMAM 5.5, PAMAM 5.5-PEG, PAMAM 6 and PAMAM 6-PEG. Blue – nuclei (DAPI), green – PAMAM (Alexa Fluor 488). Magnification × 400

As shown in Fig. 6, the actual localization of nanoparticles is difficult to assess using the microplate reader as it measures the overall fluorescence intensity of Alexa Fluor 488 conjugated – dendrimers. Visualizing fluorescently labeled nanoparticles with confocal microscopy revealed that both PAMAM types of nanoparticles without BDNF molecules coated the cell surface with similar efficacy. However, the distribution of PAMAM molecules differed between the two dendrimer types. PAMAM 5.5 aggregated around the cell surface, whereas PAMAM 6 nanoparticles were more evenly dispersed. The PEG-ylation of the dendrimer complex did not seem to affect the distribution pattern.

### Morphological alteration induced by PAMAM- and PEGylated PAMAM-based nanoparticles with differently charged PAMAM cores

**Fig. 7:**
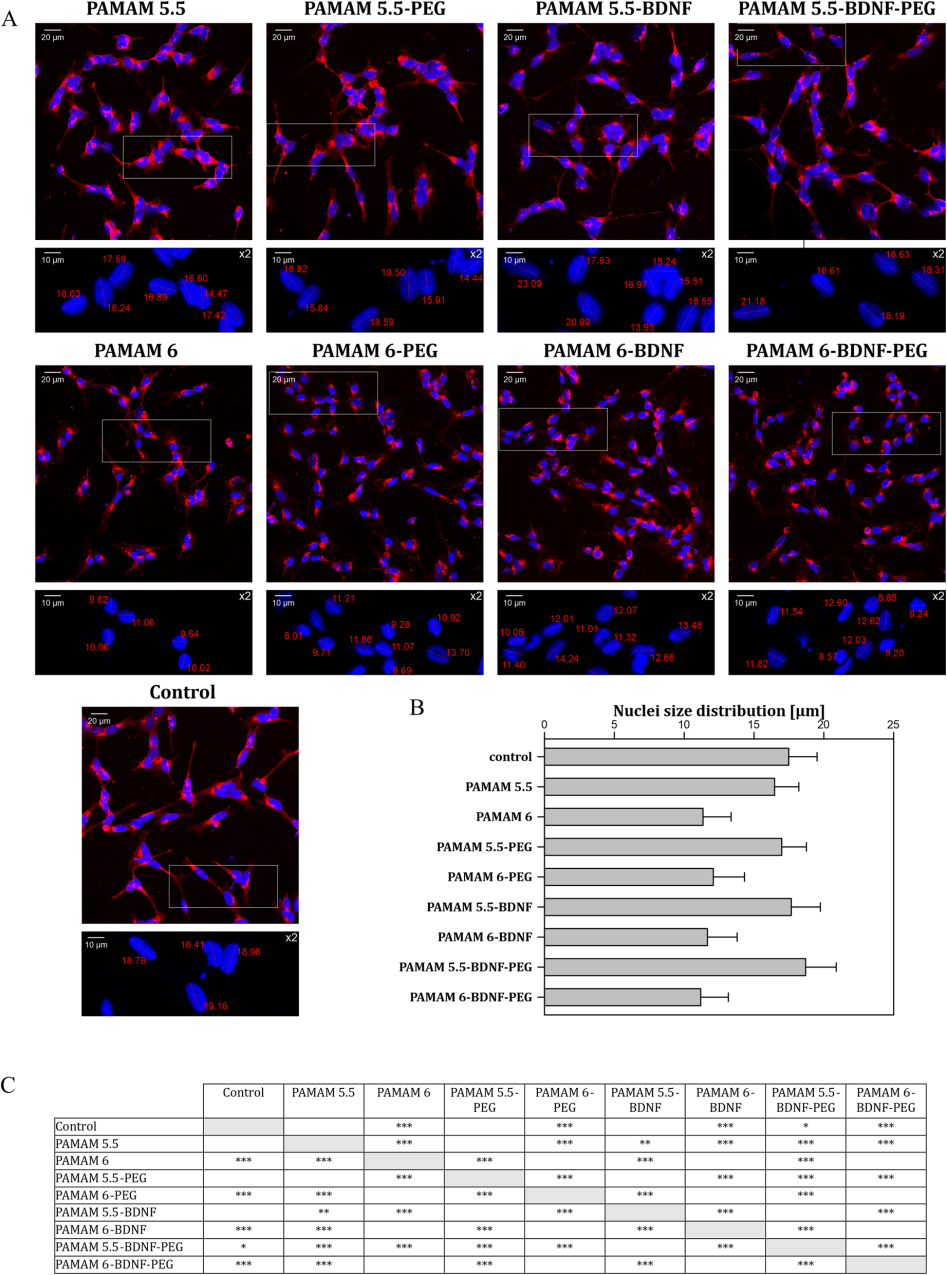
Analysis of morphological alterations in RA-differentiated 6-OHDA treated SH-SY5Y cells incubated with PAMAM 5.5, PAMAM 5.5-PEG, PAMAM 5.5-BDNF (0.1 µg/mL), PAMAM 5.5-BDNF (0.1 µg/mL)-PEG, PAMAM 6, PAMAM 6-PEG, PAMAM 6-BDNF (0.1 µg/mL) and PAMAM 6-BDNF (0.1 µg/mL)-PEG. **A** confocal images of WGA stained SH-SY5Y cells (red—surface glycoproteins; blue—nuclei), magnification × 200. A selected region has been enlarged twice and presented below each of the main pictures with marked cell nuclei measurements (presented in µm). **B** chart representing nuclei size distribution in all assessed study groups. Values are presented as mean ± SD. C: table summarizing the statistical analysis results. Calculations were performed using Kruskal–Wallis test. *p ≤ 0.05, **p ≤ 0.01, ***p ≤ 0.001

The WGA fluorescence staining was used to assess the state of differentiation of SH-SY5Y cells after the treatment with PAMAM 5.5, PAMAM 5.5-PEG, PAMAM 5.5-BDNF (0.1 µg/mL), PAMAM 5.5-BDNF (0.1 µg/mL)-PEG, PAMAM 6, PAMAM 6-PEG, PAMAM 6-BDNF (0.1 µg/mL) and PAMAM 6-BDNF (0.1 µg/mL)-PEG. The cells incubated with PAMAM 5.5, with the nanoparticle itself as well as with the PEG-ylated form, retained their differentiated shape with elongated processes (Fig. 7A). Moreover, the wheat germ agglutinin staining utilized for confocal microscopy visualization allowed us to evaluate the shape of the cells. This approach revealed that cells treated with PAMAM 5.5 stayed in their RA differentiated, elongated state, and PAMAM 6 treatment-induced alterations, which led to more round and visibly smaller cells. On the other hand, cells treated with various forms of PAMAM 6—alone, PEG-ylated, or with BDNF—presented severe phenotype alterations. These cells were round, significantly smaller, and did not show the characteristic features of neural cells compared to differentiated control or PAMAM 5.5 treated cells. Moreover, the nuclei size distribution analysis revealed a significant difference between cells incubated with differentially charged nanoparticles (Fig. 7B and C). In SH-SY5Y cells subjected to PAMAM 6 and all its tested modifications, the cell nuclei dimensions were smaller than in PAMAM 5.5 treated or control cells. Interestingly, the nuclei of SH-SY5Y cells incubated with PAMAM 5-BDNF (0.1 µg/mL)-PEG were slightly but significantly bigger than in control specimens.

### Detection of mitochondrial dysfunction and lipid peroxidation in neuroblastoma cell line after treating with PAMAM- and PEGylated PAMAM-based nanoparticles with differently charged PAMAM cores.

**Fig. 8:**
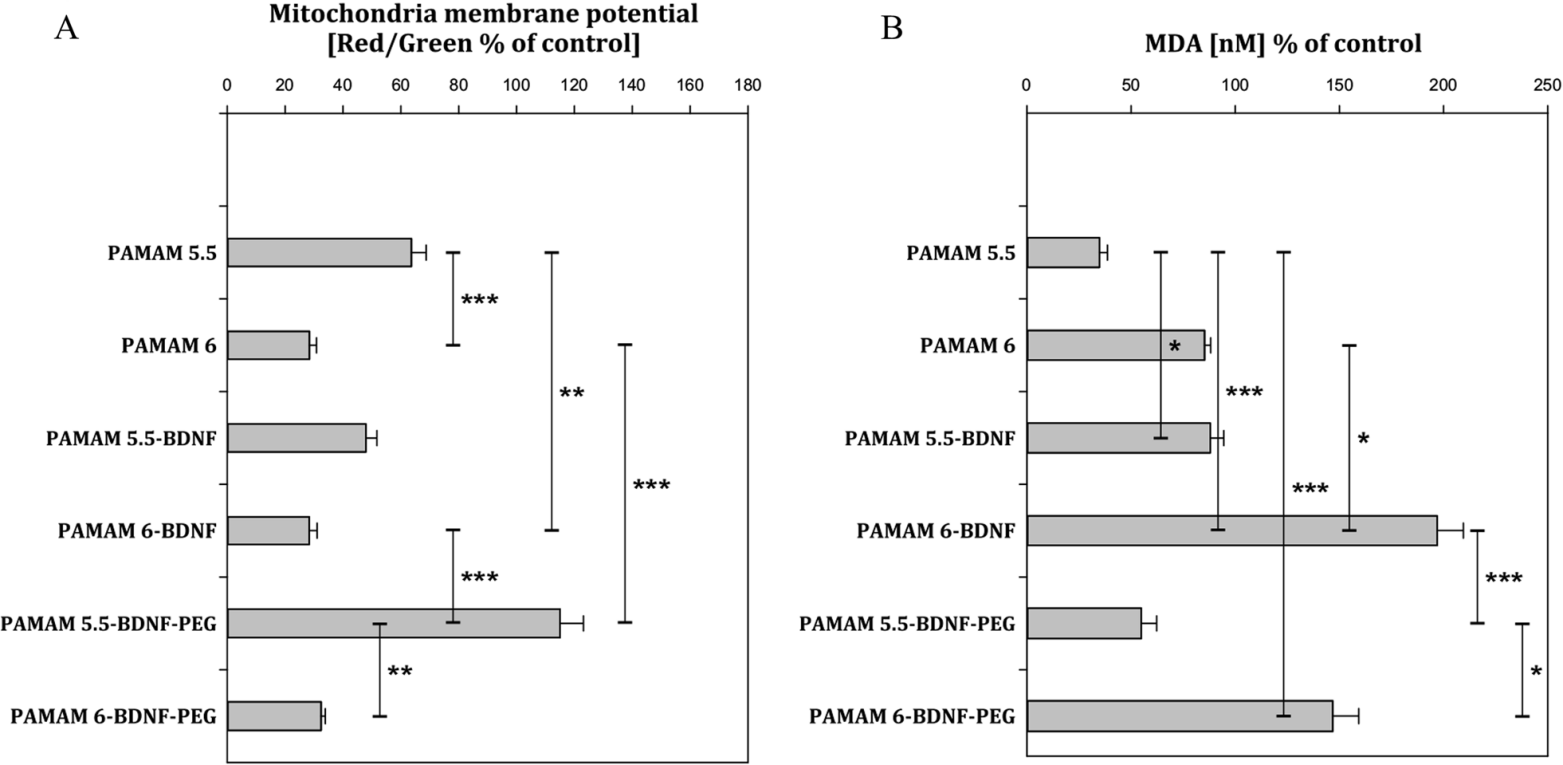
Assessment of dendrimers-based nanoparticles mitochondrial membrane potential (part A) and MDA (part B) in neuron-like differentiated human neuroblastoma cell line SH-SY5Y upon treatment for 24 h. Data is presented as the mean ± SD (*n* = 12). Data distribution was tested using Shapiro–Wilk test. To compare two analyzed datasets the unpaired t-test was used. The analysis of variances between different time-points was performed using one-way ANOVA. *—p < 0.05, **—p < 0.01, ***—p < 0.001

Based on the above results related to stimulation of neuronal-like cell proliferation and survival, we investigated whether PAMAM-based NPs can trigger oxidative damage through inducing reactive oxygen species (ROS) accumulation and if a trophic factor, BDNF, released by PAMAM-based NPs could induce oxidative stress by altering the composition of cellular membrane lipids as well as mitochondrial membrane potential of the human SH-SY5Y cell line. Several major events occur in mitochondria in response to oxidative stress, of which the most significant is the loss of mitochondrial membrane potential (MMP), leading to apoptosis. To elucidate the role of mitochondria in NPs-induced apoptosis of SH-SY5Y cells, we detected depolarization of MMP using JC-1. As shown in Fig. 8A, treatment of NPs with PAMAM 6 (for 24 h) induced significant loss of MMP, as reflected by the fluorescence shift from red to green. A decrease in the red (~ 590 nm)/green (~ 529 nm) fluorescence intensity ratio by exposure to PAMAM-based NPs indicates depolarization/disruption of the mitochondrial membrane in previously differentiated human neuroblastoma cells. PAMAM 5.5-BDNF-PEG treatment improved the MMP (120% of control MMP) in SHSY-5Y cells compared to the control (100% MMP). In contrast, all other tested dendrimers and PAMAM-based NPs can cause mitochondrial fragmentation, as demonstrated by Fig. 7A.

To further investigate the molecular mechanism of oxidative stress related to the different mitochondrial membrane depolarization found after treatment with PAMAM5.5 and PAMAM 6, we examined the concentration of MDA-(TBA)_2_ complex, as the marker of lipid peroxidation (Fig. 8B). The separation of the MDA-(TBA)_2_ complex from other interfering compounds by reverse phase HPLC technique and we observed a more pronounced reduction in MDA levels in PAMAM 5.5 and PAMAM 5.5-based nanoparticles in comparison to PAMAM6 and PAMAM6-based NPs. MDA ranges in control cells were determined to be as low as 20 nM, which is more than a fourfold increase from PAMAM 5.5. The concentrations of MDA in cell supernatant after PAMAM 6 treatment were 85 nM and increased to 198 nM for PAMAM 6 – BDNF nanoparticles. There were significant differences between PAMAM -BDNF nanoparticles compared to those with PEG layer for both generations of PAMAM dendrimers.

## Discussion

Despite long-lasting efforts with extensively studied protein adsorption using various experimental techniques, the understanding of these phenomena remains incomplete. Conflicting views persist concerning the most fundamental issues such as the nature of interactions driving the protein adsorption, reversibility, monolayer structure and stability, conformational changes, maximum coverages, and isotherm interpretation. Therefore, in this work, results are discussed pertinent to therapeutic proteins adsorption at low concentration to obtain a better efficiency of selective delivery to final tissues. This requires consideration of the most fundamental issues for protein adsorption phenomena: the forces which are driving the protein adsorption, reversibility, monolayer structure and stability, degree of conformational changes, and maximum coverages, etc. To understand more complex processes with protein interactions at in vitro level, one may suppose that designing of nanoparticles to approach their destination can be improved if the following aspects are carefully addressed: physicochemical characteristics of protein molecule solutions, such as the concentration, aggregation degree, purity, size and shape (actual conformation), charge, isoelectric point, stability of the solutions, etc. should be determined before performing adsorption experiments. The influence of the supporting electrolyte composition, its ionic strength, pH (buffers) on the above parameter should also be investigated. The substrates' specific characteristics in adsorption experiments, primarily their chemical composition, homogeneity, geometrical topology, roughness, charge distribution, stability, etc., are also prerequisites. Recently, various compounds and preparation methods have been explored to improve stability and water-solubility of polymeric drug delivery systems, which enable sustained and efficient delivery of therapeutic protein over prolonged periods, leading to the formulation of DNA [37], chitosan, polyelectrolytes [38], pyrrole, silica, gold, cells, and dendrimer-based nanoparticles. Cytotoxicity of the active core of NP and its poor cellular internalization is among the most critical factors that contribute to unfavourable side effects caused by noneffective active compound delivery. Despite the large number of studies [18, 39, 40], the application of dendrimers in biomedicine is rather difficult due to a lack of understanding of the relationship between physicochemical properties of protein-based dendrimer NP and the mechanism of their cellular uptake and cytotoxicity. In the present study, we focused on the electrokinetic charge of PAMAM dendrimers in constructing (PEGylated)PAMAM-BDNF nanoparticles using a physicochemical approach which constitutes the most significant parameter affecting PAMAM-based nanoparticles in vitro toxicity, biopermeability, and oxidative stress properties.

It has been reported that charged ligands can prevent or enhance linking chemistry via electrostatic repulsion or attraction and dramatically affect nonspecific interaction/adsorption [40–42]. We found that NPs with tailored charge properties could allow/improve the protection of BDNF from nonspecific binding with serum proteins and could enable controlled delivery of BDNF in two manners: (1) indirectly via reversibly adsorbed BDNF molecules at NPs surface or (2) directly via NPs and irreversibly bound BDNF. A similar finding was proposed for another nanoscale complex of polymer poly(ethylene glycol)-*b*-poly(L-glutamic acid) with BDNF protein, where molecular dynamics simulations suggest binding between BDNF and PEG-PLE is mediated through electrostatic coupling as well as transient hydrogen bonding. In this study, Yuhang Jiang et al. suggested that poor intranasal-to-brain bioavailability of BDNF can be attributed to its cationic surface charges and extensive binding to various polysaccharides, resulting in its entrapment and degradation within the negatively charged tissues [35].

In our study, the electrophoretic mobility of the BDNF-PAMAM nanoparticles was much lower than the electrophoretic mobility of bulk protein, which corresponded to the formation of an unsaturated BDNF layer on PAMAM as well as structural alterations/conformation change of BDNF that can be induced upon NP-binding. It has been shown by various theoretical calculations performed using the chemical composition of proteins that the surface charge of the molecule is heterogeneously distributed in the form of positive and negative patches, existing over a wide range of pH. This hypothesis is supported by comparable results reported in the literature on proteins: HSA [43], IgG [44], fibrinogen [45], NT-4 [34], and lysozyme [46].

We carried out BDNF adsorption at PAMAM dendrimers in high ionic strength (0.15 M) solution because the reduction of the repulsive interactions between the protein and polymers has been previously reported for different proteins, including polyclonal rabbit immunoglobulin (IgG) [47], fibrinogen [45], albumin [43], and NT-4 [34]. By applying this approach, we obtained a higher adsorbed amount of BDNF which makes our nanocarriers more biocompatible.

Our previous study [48] showed that the maximum coverage of the irreversibly bound protein increases monotonically with increasing ionic strength—this directly translates to our current study, in which the range between BDNF and PAMAM molecules, determining lateral electrostatic interaction, decreases proportionally to the double layer thickness (according to Debye length: 1/(kappa *a*)^½^ (a is a characteristic protein dimension and κ^−1^ Debye screening length)), which is correlated with ionic strength of a solution. One can speculate that this effect is caused by additional interactions, most likely of the van der Waals type or hydrogen bonding described elsewhere [49, 50].

Another contribution to attractive interactions at this pH may originate from the heterogeneous charge distribution over the BDNF molecule, which in our study was suggested by a slightly positive charge of protein at pH 7.4. Therefore, even if the overall charge of the BDNF molecule remains positive, there are patches bearing a negative charge. It should be mentioned that anomalous protein deposition under physiological conditions was previously observed for various proteins e.g., fibrinogen by Wasilewska and Adamczyk [29], Malmsten [30], Ortega-Vinuesa et al. [31], Toscano and Santore [32], albumins and neurothrophins [33]. Based on the abovementioned findings, we show that BDNF adsorption at the PAMAM dendrimers with anionic (sodium carboxylate) and cationic (amine) terminal groups is significant and consistent with a binding mechanism that involves weak interactions between dendrimer terminal groups and the BDNF amino acids residues*.* Our observation of these effects of dendrimer terminal group chemistry on the protein adsorption on PAMAM dendrimers is in accordance with the results reported by Bo Wang et al. [41], where areas of HSA with many acidic residues were attached to the positively charged binding sites on PAMAM. We observed a low residual protein concentration after adsorption, reflecting that for negatively charged PAMAM the BDNF amino acid residues are slightly protonated, positively charged, and attached to PAMAM, whereas for positively charged PAMAM the BDNF amino acid residues are slightly deprotonated, negatively charged and attached to PAMAM. The effect was more pronounced for negatively charged dendrimers than positively charged one. This suggests that electrostatic interactions are crucial in binding charged dendrimers with proteins, which has also been observed for chymotrypsin, IgE [41], and cytochrome-c [51].

Moreover, one can see that for both tested protein concentrations at PAMAM 5.5 dendrimer surface, desorption of protein molecule was negligible, indicating that its adsorption onto 10 mgL^−1^ PAMAM nanoparticles was almost completed. This way, we found that PAMAM molecules for laden BDNF concentrations of fewer than 1 mgL^−1^ are likely to form an irreversibly adsorbed BDNF layer. Therefore, the effect of the substrate remains significant, making the desorption process less efficient than in the case of negatively charged dendrimer core. It should be mentioned that these results correspond to the irreversibly adsorbed fraction of protein molecules forming an intimate contact with interfaces and characterized by the binding energy smaller than −20 *kT*  [52]. We determined that the coverage of BDNF molecules at the dendrimer surface is independent of the bulk neurotrophin concentration and transport mechanism.

Furthermore, we noticed that the adsorption of BDNF molecules on dendrimer nanocarrier, regardless of its surface electrokinetic charge, prevents protein aggregation and thus could enable interaction with the receptors, making them more biocompatible and suitable for a range of biomedical applications. Yuhang J. et al. also observed that the BDNF self-assembles with anionic block copolymers into nanosized complexes, stabilized by cooperative electrostatic interaction, and H-bonds were nearly monodispersed at specific charge ratios [35]. These findings are in good agreement with previous studies for the interaction of dendrimer with human serum albumin [53] and intracellular ion [54].

The surface chemistry of dendrimers has been found to influence their cytotoxicity, which varies between cell types as well as dendrimers functionalization with –OH, –COOH, –NH_2_ terminal groups, PEG-ylation [55, 56]. The variations in cell response found in cytotoxicity studies can be determined by different physicochemical properties of PAMAM dendrimers and their interactions with cell membranes which remains poorly understood [18]. The cytotoxicity of PAMAM dendrimers as a result of the interactions between positively charged dendrimers and negatively charged protein or cell surface was described by Wei Wang et al. [39] That findings are in good agreement with our results that strongly suggest that electrostatic interaction of positively charged PAMAM 6 dendrimers with cell membranes decreases their viability as well as increases phenotype alterations in comparison to negatively charged PAMAM 5.5 dendrimers.

It should be underlined that determination of the cellular localization of PAMAM and PEG-PAMAM nanoparticles reveals aggregation processes in an in vitro environment. This was more apparent for negatively charged PAMAM dendrimer (without BDNF) than positively charged PAMAM dendrimer (without BDNF), which strongly suggests that more monodispersed dendrimer molecules can easily interact with the negatively charged cell surface. The experiments demonstrated that occurring electrostatic interactions play a significant role in the PAMAM interaction with cell surfaces. Above mentioned experiments also revealed that encapsulation in the polyelectrolyte layer did not influence the adsorption efficiency of nanoparticles on the cell membrane. It seems that PEG's floppy chains and their charge neutrality cannot prevent non-specific adsorption of nanoparticles in in vitro environment. PEG-ylation could prevent or enhance linking chemistry between PAMAMs via electrostatic repulsion or attraction, which are necessary to produce adequate binding energy to create aggregates in differentiated human neuroblastoma cell line culture SH-SY5Y treatment for 24 h.

We also had shown that there was only a minor change in cell viability or phenotype alteration of tested cells when they were exposed to a modified PAMAM 6 dendrimers (initially positively charged) by adsorption of BDNF or PEG even if the charged groups on the surface of the dendrimers was neutralized and gave nominally uncharged NP. In our study, after PEG adsorption at negatively charged PAMAM-BDNF nanoparticles, the zeta potential of saturated PEG layers displayed a negative value. This suggests that the surface at the brush base is net negative and that the shear plane for zeta potential study lies substantially beneath the brush height. This could be related to a shift of the hydrodynamic phase of shear to lower distances of the surface of the nanoparticles. Similar observations have been previously described elsewhere [57–59].

It has been established that dendrimers’ cytotoxicity depends on the generation and concentration of the dendrimer and varies with cell types [18, 60]. One of the key factors that determining the toxicity of several nanomaterials, including dendrimers, is oxidative stress [61, 62]. Reactive oxygen species (ROS) formation causes damage to biological components through oxidation of lipids, proteins, and DNA damage, finally leading to apoptosis [63]. Mukherjee et al. have proposed that the cationic PAMAM dendrimers enter the cell via endocytosis and are transported in endosomes and localize in mitochondria [64]. Next, the dendrimers induce an increase of internal mitochondrial pH due to an acid–base equilibrium reaction between secondary amines and their conjugate base, resulting in ROS production. Our study found significantly increased levels of malonyl dialdehyde (MDA) after PAMAM6-based nanoparticles administration compared to the PAMAM5.5-ones. This further supports this notion of positively charged PAMAM6 effects on ROS generation, as MDA is an end-product of PUFAs (polyunsaturated fatty acids) oxidation. It serves as an indicator of lipid peroxidation and oxidative stress [65]. Interestingly, we observed that the functionalization of PAMAM dendrimers with PEG reduces MDA concentration. The reduction of cytotoxicity with PEG addition has been previously described by several groups and is generally attributed to changes of dendrimer surface charge that influence interactions with cellular membranes [66, 67].

Oxidative damage is also linked with mitochondrial dysfunction, which includes changes in the membrane potential and alterations to the oxidation–reduction potential of the mitochondria [68]. In our study, we found that the detrimental cellular effect of PAMAM6-based NPs administration was also reflected in the induction of mitochondrial depolarization. In contrast, exposure to PAMAM 5.5-BDNF-PEG induced a significant increase in mitochondrial membrane potential. Nyitrai et al. have described a prominent mitochondrial membrane depolarization in pyramidal neurons and astroglial rat cells after exposure to PAMAM5, which they have attributed to the enhancement of intracellular Ca^2+^ level [69]. Interestingly, when dendrimer was administered together with Ca^2+^, the protective effects against mitochondrial depolarization caused by calcium ions was observed [70]. As Janaszewska et al. have noted, PAMAM functionalization (in that case with 4-carbomethoxypyrrolidone) resulted in a lack of influence of the PAMAM-pyrrolidone dendrimer on intracellular ROS level and mitochondrial membrane potential [60]. This suggests that our results of improved MMP in the case of PAMAM5.5-PEG could be attributed to the beneficial influence of PEG on the nanoparticle surface, which results in a favorable effect on mitochondrial membrane potential.

## Conclusions

We provide a characterization of efficient encapsulation of BDNF into PEG-ylated-PAMAM complex with an anionic or cationic core charge, with a particular focus on electrostatic interactions in the constructed nanoparticles and their behavior in differentiated human neuroblastoma cell line SH-SY5Y treated with 6-OHDA. The key finding of our study was that PAMAM5.5 and PAMAM6 dendrimer-based nanoparticles differ significantly in terms of BDNF release profile from their surfaces and their effect on cell viability, mitochondrial membrane potential, cell phenotype, and induction of oxidative stress. Our results confirm that the design of nanoparticles for biological applications requires a thorough understanding and consideration of electrostatic interactions between components of the nanoparticle.

## Supporting information

Determination of the cytotoxicity of 6-OHDA on differentiated human neuroblastoma SH-SY5Y cells after 24 h of incubation with the flow cytometry and MTT assay.

## Supplementary Information

**Additional file 1**: **Figure S1**. Cytotoxicity curves for various concentration of 6-OHDA in differentiated human neuroblastoma cell line SH-SY5Y: a) MTT assay, b) Flow cytometry. The data represent means +/- SD for 20 experiments.

## Data Availability

The data required to reproduce these findings are availability for any research.
