# Supplementary material for: The role of the electrokinetic charge of neurotrophis-based nanocarriers: protein distribution, toxicity, and oxidative stress in in vitro setting
Source: J Nanobiotechnology. 2021 Aug 28;19:258. doi: 10.1186/s12951-021-00984-4 (PMC8399784; doi:10.1186/s12951-021-00984-4)
Supplement: Supplementary file 1 — Additional file 1: Figure S1. Cytotoxicity curves for various concentration of 6-OHDA in differentiated human neuroblastoma cell line SH-SY5Y: a) MTT assay, b) Flow cytometry. The data represent means +/- SD for 20 experiments. [file 12951_2021_984_MOESM1_ESM.docx]

**SUPPORTING INFORMATION**

**The role of the electrokinetic charge of neurotrophis-based nanocarriers: protein distribution, toxicity, and oxidative stress in *in vitro* setting**

# Maria Dąbkowska^1^*, Zofia Ulańczyk^2^, Karolina Łuczkowska^2^, Dorota Rogińska^2^, Anna Sobuś^2^, Monika Wasilewska^3^, Maria Olszewska^1^, Katarzyna Jakubowska^4^, Bogusław Machaliński^2^

^1^*Department of Medical Chemistry,* ***Pomeranian Medical University, Rybacka 1, 70-204*** *Szczecin, Poland*.

*^2^Department of General Pathology,* ***Pomeranian Medical University, Rybacka 1, 70-204*** *Szczecin, Poland.*

*^3^Jerzy Haber Institute of Catalysis and Surface Chemistry Polish Academy of Sciences, Niezapominajek 8, 30-239 Cracow, Poland.*

*^4^Department of Biochemistry,* ***Pomeranian Medical University, Rybacka 1, 70-204*** *Szczecin, Poland.*

*Corresponding author

Maria Dąbkowska

Department of Medical Chemistry,

**Pomeranian Medical University,**

**Rybacka 1, 70-204** Szczecin, Poland

maria.dabkowska@pum.edu.pl

1. Determining the cytotoxicity of 6-OHDA with the MTT assay.

Cytotoxicity of 6-OHDA was evaluated by exposing cells to different concentrations of this neurotoxin for 24h at 37^o^C. Cell viability was estimated by measuring toxicity using the MTT assay and flow cytometry.

We established that exposure to 20 µmol/L doses of 6-OHDA resulted in 20% decline in cell viability, which resulted in the damaging of differentiated human neuroblastoma SH-SY5Y cells. The experimental results obtained from the MTT assay/flow cytometry are presented in Fig. S1.

a)


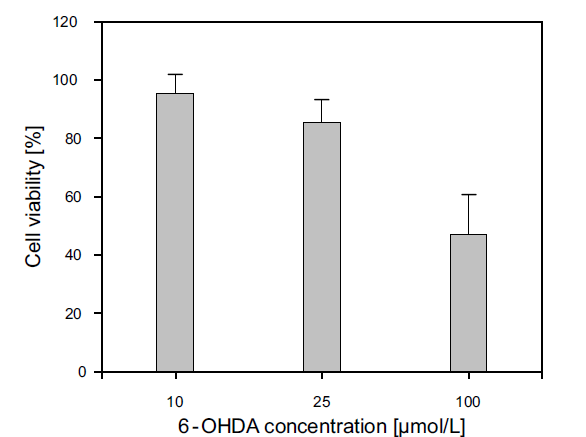


b)


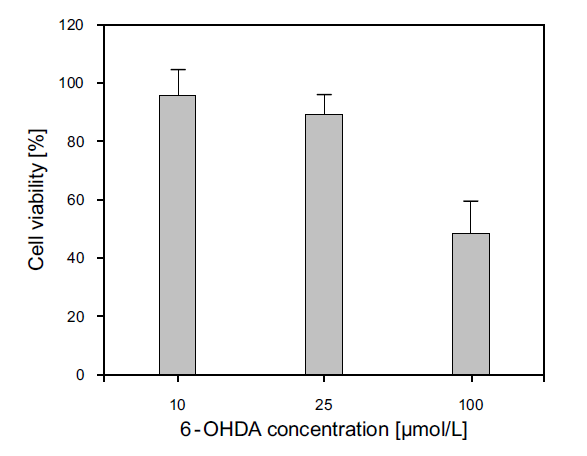


**Fig. S1.** Cytotoxicity curves for various concentration of 6-OHDA in differentiated human neuroblastoma cell line SH-SY5Y: a) MTT assay, b) Flow cytometry. The data represent means +/- SD for 20 experiments.
